# Editorial: Environmental impacts on women’s health disparities and reproductive health: advancing environmental health equity in clinical and public health practice

**DOI:** 10.3389/frph.2024.1484406

**Published:** 2024-10-23

**Authors:** Melissa M. Smarr, Kristen M. Rappazzo, Darlene Dixon

**Affiliations:** ^1^ Population Health Branch, Division of Extramural Research and Training, National Institute of Environmental Health Sciences, Durham, NC, United States; ^2^ U.S. Environmental Protection Agency Office of Research and Development, Center for Public Health and Environmental Assessment, Research Triangle Park, Durham, NC, United States; ^3^ Molecular Pathogenesis Group, Mechanistic Toxicology Branch, Division of Translational Toxicology, National Institute of Environmental Health Sciences, National Institutes of Health, Durham, NC, United States

**Keywords:** environmental health disparities, women’s reproductive health, environmental justice, reproductive health equity, place-based factors

**Editorial on the Research Topic**
Environmental impacts on women’s health disparities and reproductive health: advancing environmental health equity in clinical and public health practice

Cumulative impacts of chemical and social factors challenge progress toward achieving equity and justice in the context of environmental exposures and somatic health, including reproductive well-being. As we work toward achieving justice in women's reproductive health, this Research Topic highlights studies that are designed to closely examine upstream factors to identify why disparate exposures and disproportionate adverse reproductive outcomes exist. Our intention for this editorial is to reiterate our continuing commitment to achieving environmental health equity and to call attention to the research impacts and lessons learned by the presenters at the National Institute of Environmental Health Sciences (NIEHS)-hosted workshop, “Environmental Impacts on Women's Health Disparities and Reproductive Health”, held on 27–28 April 2022 (https://www.niehs.nih.gov/news/events/pastmtg/2022/ehdworkshop2022/index.cfm). The purpose of the workshop was to place emphasis on research examining the effects of chemical and non-chemical stressors on adverse maternal and fetal health outcomes, to discuss diseases specific to women and individuals assigned female at birth, and to assess the role of racial and ethnic disparities in environmental exposures.

We strongly encourage readers to thoughtfully consider the novel concepts proposed for conducting health disparity research to achieve health equity and environmental justice, the lessons learned, and the general knowledge gleaned from the workshop presentations, some of which are included in this Research Topic.

• To address health disparities, achieve health equity, and advance environmental justice it is essential that the research begins to closely examine upstream factors to identify why these disparate exposures and disproportionate adverse reproductive outcomes exist.
• To elucidate the role of the environment in reproductive health disparities, a shift is needed from the traditional concept of ‘environment’ to a contemporary lens that includes the built environment and place-based factors.
• Increasing research efforts toward translational environmental epidemiologic research frameworks, transdisciplinary community driven, comprehensive research that leads to action and informs policy will advance environmental health equity.
• The development of novel measures or the use of existing measures that other disciplines utilize to assess structural racism will be key as we continue to work toward achieving justice in reproductive health. Additionally, this means that there is room to be creative and inclusive of qualitative and mixed-method approaches to achieving equity in reproductive health.
• Most importantly, and often overlooked, is the need to create equal access to tools and opportunities to improve environmental health equity.

The selected papers in this Research Topic demonstrate the commitment that many in the field of environmental health sciences have made toward elucidating the intersecting systems that impact and marginalize many racial and ethnic populations at various stages of their life course. Specific examples of articles in this Research Topic include an assessment of racial/ethnic and educational differences in menstrual and intimate care product use among people who menstruate; a causal mediation analysis to examine whether racial and ethnic disparities in preterm birth may be partially explained by exposure to a class of chemicals used as flame retardants in the United States (polybrominated diphenyl ethers); the place-based impacts of environmental justice burdens (i.e., a neighborhood characterized by both increased environmental burden and socioeconomic deprivation) on racial disparities in spontaneous preterm birth; and investigating racial/ethnic differences in household food security status in the context of cardiometabolic health among pregnant people in the United States. Furthermore, some work focused on reproductive outcomes in offspring—racial disparities in the association between gestational exposure to a mixture of phthalates and fetal genital development.

We encourage readers of this special issue to consider their commitment to pursuing environmental and reproductive health equity. Specifically, to be intentional with the design of research studies to carefully consider the role of limited educational and employment opportunities; reduced residential options and hazardous residential characteristics; systemic barriers (e.g., redlining) and discriminatory policies (e.g., urban sprawl) that may increase the risk of adverse reproductive health outcomes.

